# Sweroside Ameliorated Memory Deficits in Scopolamine-Induced Zebrafish (*Danio rerio*) Model: Involvement of Cholinergic System and Brain Oxidative Stress

**DOI:** 10.3390/molecules27185901

**Published:** 2022-09-11

**Authors:** Ion Brinza, Mohamed A. El Raey, Walaa El-Kashak, Omayma A. Eldahshan, Lucian Hritcu

**Affiliations:** 1 Department of Biology, Faculty of Biology, Alexandru Ioan Cuza University of Iasi, Bd. Carol I, No. 11, 700505 Iasi, Romania; 2 Department of Phytochemistry and Plant Systematics, National Research Centre, Dokki, Cairo 12622, Egypt; 3 Department of Chemistry of Natural Compounds, National Research Centre, Dokki, Cairo 12622, Egypt; 4 Department of Pharmacognosy, Faculty of Pharmacy, Ain Shams University, Abbassia, Cairo 11566, Egypt; 5 Center of Drug Discovery Research and Development, Ain Shams University, Abbassia, Cairo 11566, Egypt

**Keywords:** sweroside, *Schenkia spicata*, scopolamine, zebrafish, memory, cholinergic function, oxidative stress

## Abstract

Sweroside is a secoiridoid glycoside and belongs to a large group of naturally occurring monoterpenes with glucose sugar attached to C-1 in the pyran ring. Sweroside can promote different biological activities such as antifungal, antibacterial, hepatoprotective, gastroprotective, sedative and antitumor, antioxidant, and neuroprotective activities. Zebrafish were given sweroside (12.79, 8.35, and 13.95 nM) by immersion once daily for 8 days, along with scopolamine (Sco, 100 μM) 30 min before the initiation of the behavioral testing to cause anxiety and memory loss. Employing the novel tank diving test (NTT), the Y-maze, and the novel object recognition test (NOR), anxiety-like reactions and memory-related behaviors were assessed. The following seven groups (*n* = 10 animals per group) were used: control, Sco (100 μM), sweroside treatment (2.79, 8.35, and 13.95 nM), galantamine (GAL, 2.71 μM as the positive control in Y-maze and NOR tests), and imipramine (IMP, 63.11 μM as the positive control in NTT test). Acetylcholinesterase activity (AChE) and the antioxidant condition of the brains were also evaluated. The structure of sweroside isolated from *Schenkia spicata* was identified. Treatment with sweroside significantly improved the Sco-induced decrease of the cholinergic system activity and brain oxidative stress. These results suggest that sweroside exerts a significant effect on anxiety and cognitive impairment, driven in part by the modulation of the cholinergic system activity and brain antioxidant action.

## 1. Introduction

Alzheimer’s disease (AD), which makes up the majority of dementia cases, is a neurodegenerative disease that is defined by a deterioration in cognitive function and a progressive loss of memory due to a decrease in hippocampus neurogenesis [1,2]. AD pathophysiology also includes neuroinflammation, oxidative stress, decreased synaptic plasticity, and cholinergic dysfunction [3]. Acetylcholine (ACh) levels in the hippocampus have been raised by cholinergic medications in clinical studies as a treatment for AD [4]. Compounds made from natural substances that simultaneously target numerous diseased components may be a potential approach for the safer treatment of AD.

Historically, natural products have played a vital role in the discovery of many drugs and contributed considerably to pharmacotherapy. Natural extracts and compounds showed a wide range of activities against many disorders [5,6,7]. An important phytochemical is sweroside which is a secoiridoid glycoside. It belongs to a large group of naturally occurring monoterpenes with glucose sugar attached to C-1 in the pyran ring [8]. Sweroside is a bioactive herbal ingredient, which has diverse biological activities.

In LPS-induced RAW264.7 cells, sweroside could reduce inflammation by inhibiting cell proliferation, reducing pro-inflammatory cytokines, and increasing anti-inflammatory cytokines while stopping the cell cycle (at the G0/G1 phase). Additionally, it can activate sirtulin 1 (SIRT1), inhibit nuclear factor-kappa B, and enhance Forkhead transcription factor O1 signaling pathways [9].

Human leukemia cell lines and primary human leukemia cells had lower cell viability after treatment with sweroside. S and G2/M cell-cycle arrest was elicited. Additionally, sweroside significantly increased the levels of cleaved caspase-3 and poly (ADP-ribosyl) transferase (PARP), which in turn increased the amount of apoptosis both in vitro and in vivo. It could prevent the formation of HL-60-carrying tumors by inducing apoptosis and suppressing proliferation [10]. This compound was able to regulate lipid metabolism and inflammatory responses via the regulation of peroxisome proliferator-activated receptors (PPAR)-α in fatty livers [11]. It attenuated alpha-naphthylisothiocyanate (ANIT)-induced cholestatic liver injury in mice by restoring bile acid synthesis and bringing it to its normal levels [12]. It regulated the phosphoenolpyruvate carboxykinase (Pck1) expression and induced insulin-mimicking effects [13].

Sweroside affects the osteoblastic MC3T3-E1 cells by interacting with the membrane estrogen receptor-*α* and GPR30, which subsequently activates the p38 signaling pathway, and is thought to be a viable therapy for osteoporosis [14]. 

In the current study, we aimed to investigate the beneficial effects of sweroside in a Sco-induced zebrafish model. Because Sco is a non-selective muscarinic acetylcholine receptor blocker, it hinders learning and short-term memory, which facilitates amnesia [15]. According to reports, Sco-induced amnesia causes the brain to experience more oxidative stress [16]. We examined the antiamnesic and antioxidant properties of sweroside obtained from the *Schenkia spicata* against Sco using several methods (NTT, Y-maze, and NOR tests) with zebrafish (*Danio rerio*).

## 2. Results and Discussion

### 2.1. Effects on Anxiety-Like Behavior in the NTT Test

**Figure 1:**
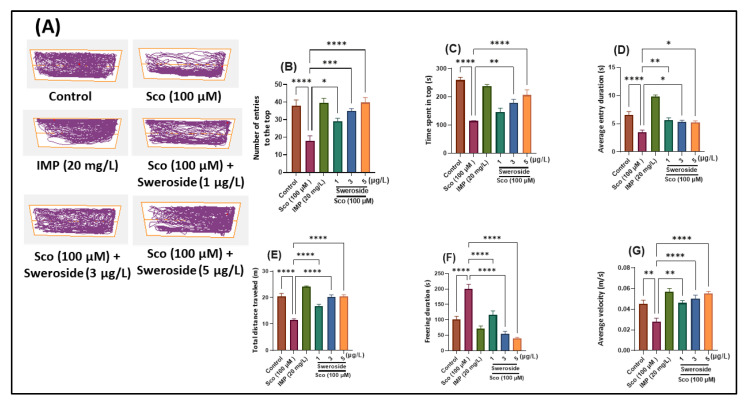
NTT results for sweroside (2.79, 8.35, and 13.95 nM). (**A**) Representative tracking locomotion patterns in various groups; (**B**) Number of entries to the top; (**C**) Time spent in top (s); (**D**) Average entry duration (s); (**E**) Total distance traveled (m); (**F**) Freezing duration (s); and (**G**) Average velocity (m/s). Data are presented as means ± S.E.M. (*n* = 10 animals per group). * *p* < 0.01, ** *p* < 0.001, *** *p* < 0.0001, and **** *p* < 0.00001 (Tukey’s post hoc analyses). Imipramine (IMP, 63.11 μM) was used as a reference positive drug.

Figure 1 depicts the primary effects of Sco (100 µM) and sweroside (2.79, 8.35, and 13.95 nM) treatment on the anxiety response in the NTT test. Figure 1A shows how the locomotor tracking behaviors of distinct groups of zebrafish at the top and bottom zones of the tank vary. The one-way ANOVA revealed that the treatment had significant overall effects on the number of entries to the top [F (5, 54) = 11.09] (*p* < 0.0001) (Figure 1B), time spent in top [F (5, 54) = 21.78] (*p* < 0.0001) (Figure 1C), average entry duration [F (5, 54) = 31.57] (*p* < 0.0001) (Figure 1D), total distance traveled [F (5, 54) = 38.98] (*p* < 0.0001) (Figure 1E), freezing duration [F (5, 54) = 31.43] (*p* < 0.0001) (Figure 1F), and average velocity [F (5, 54) = 11.39] (*p* < 0.0001) (Figure 1G).

In comparison to the control group, the Sco-treated group displayed fewer entries to the top of the tank (*p* < 0.00001) (Figure 1B) and spent less time in the top zone of the tank (*p* < 0.00001) (Figure 1C) and displayed a shorter average entry duration (*p* < 0.00001) (Figure 1D). Additionally, Sco exposure reduced locomotion, as seen by decreased total distance traveled (*p* < 0.00001) (Figure 1E), increasing freezing duration (*p* < 0.00001) (Figure 1F), and lower average velocity (*p* < 0.001) (Figure 1G). Sweroside administration also increased the number of entries to the top zone of the tank in a dose-dependent manner (*p* < 0.01 for 2.79 nM, *p* < 0.0001 for 8.35 nM, and *p* < 0.00001 for 13.95 nM) (Figure 1B) and increased the time spent there in a dose-dependent manner (*p* < 0.001 for 8.35 nM and *p* < 0.00001 for 13.95 nM) (Figure 1C), while decreasing the average entry duration (*p* < 0.001 for 2.79 nm, *p* < 0.01 for 8.35 nM, and *p* < 0.01 for 13.95 nM) (Figure 1D). Additionally, when compared to zebrafish treated with Sco alone, zebrafish exposed to sweroside significantly altered their locomotor activity by increasing the total distance traveled in the tank (*p* < 0.00001 for 2.79, 8.35, and 13.95 nM) (Figure 1E), decreasing freezing duration (*p* < 0.00001 for 2.79, 8.35, and 13.95 nM) (Figure 1F), and increasing the average velocity (*p* < 0.001 for 2.79 nM and *p* < 0.00001 for 2.79 nM and 13.95 nM) (Figure 1G).

Previous data indicated the anxiolytic-like effects of sweroside treatment, which agree with the results from this investigation. Mahendran et al. [17] reported that methanolic extract of *Swertia corymbose* exhibited an anxiolytic profile in Swiss mice mainly attributed to the presence of swertiamarin, sweroside, and gentiopicroside.

### 2.2. Effects on Response to Novelty and Recognition Memory in the Y-maze and NOR Tests

**Figure 2:**
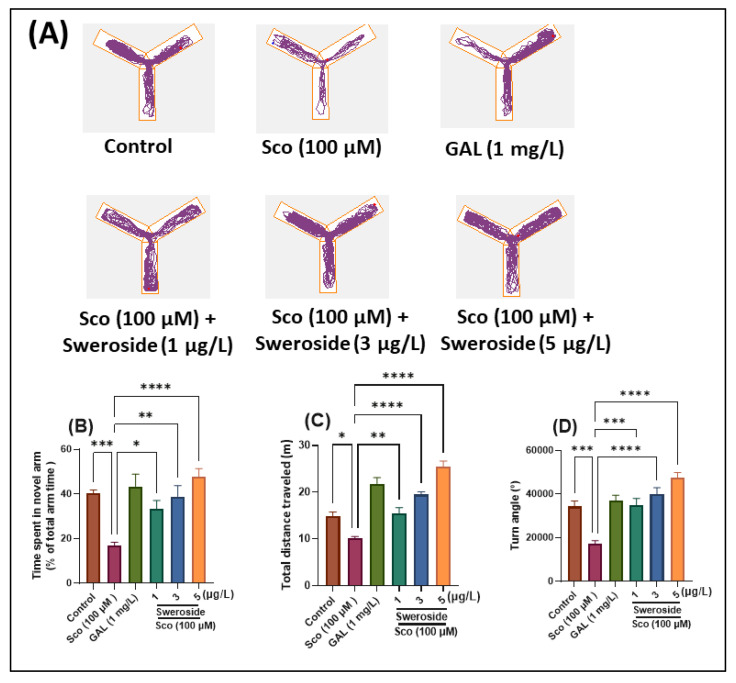
Assessment of response to novelty in Sco-treated zebrafish subjected to sweroside treatment at doses of 2.79, 8.35, and 13.95 nM in the Y-maze test. (**A**) Representative tracking locomotion patterns in various groups; (**B**) Time spent in the novel arm (% of total arm time); (**C**) Total distance traveled (m); (**D**) Turn angle (°). Data are presented as means ± S.E.M. (*n* = 10 animals per group). * *p* < 0.01, ** *p* < 0.001, *** *p* < 0.0001, and **** *p* < 0.00001 (Tukey’s post hoc analyses). Galantamine (GAL, 2.71 μM) was used as a reference positive drug.

To assess the effect of sweroside on the response to novelty, zebrafish were assessed by Y-maze for time spent in the novel arm. Figure 2A depicts typical zebrafish locomotor tracking patterns for various groups. The one-way ANOVA showed that the treatment had a substantial overall impact on the time spent in the novel arm [F (5, 54) = 7.96] (*p* < 0.0001) (Figure 2B), total distance traveled [F (5, 54) = 32.31] (*p* < 0.0001) (Figure 2C), and turn angle [F (5, 54) = 14.66] (*p* < 0.0001) (Figure 2D). Zebrafish who had received the Sco treatment spent much less time in the novel arm than the control group did (*p* < 0.0001). In contrast to the rats treated with Sco alone, sweroside significantly reversed this amnesic effect with *p* values of 0.01 for 2.79 nM, 0.001 for 8.35 nM, and 0.0001 for 13.95 nM. According to a significant reduction in the total distance traveled (*p* < 0.01) and the turn angle (*p* < 0.0001), as compared to the control group, zebrafish exposed to Sco exhibited reduced locomotor activity. In addition, sweroside treatment in Sco-induced zebrafish increased locomotion in the Y-maze by increasing total distance traveled *p* < 0.001 for 2.79 nM, *p* < 0.00001 for 8.35 nM, and *p* < 0.00001 for 13.95 nM as well as turn angle with *p* < 0.0001 for 2.79 nM, *p* < 0.00001 for 8.35 nM, and *p* < 0.00001 for 13.95 nM.

**Figure 3:**
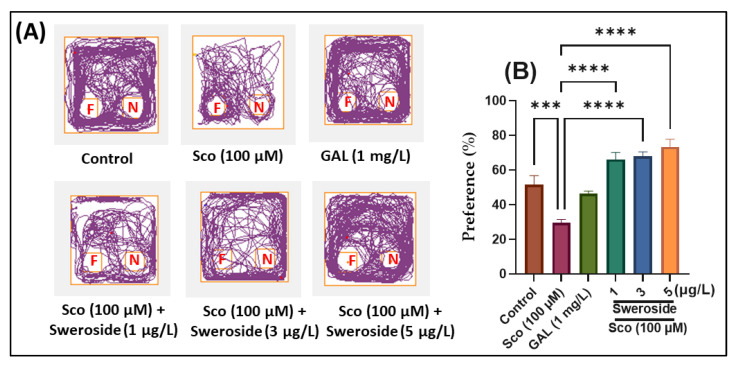
Evaluation of recognition memory in Sco-treated zebrafish subjected to sweroside treatment at doses of 2.79, 8.35, and 13.95 nM in the NOR test: (**A**) Representative tracking locomotion patterns in various groups; (**B**) Preference (%). Data are presented as means ± S.E.M. (*n* = 10 animals per group). *** *p* < 0.0001 and **** *p* < 0.00001 (Tukey’s post hoc analyses). Galantamine (GAL, 2.71 μM) was used as reference positive drug.

In the NOR test, the animal’s ability to distinguish a novel object from a familiar object was also evaluated. Figure 3A depicts typical zebrafish locomotor tracking patterns for various groups. One-way ANOVA results showed that the treatment had substantial overall impacts on the percentage of preference [F (5, 54) = 21.53] (*p* < 0.0001) (Figure 3B). Zebrafish given Sco had trouble distinguishing between unfamiliar and familiar objects, which led to a substantial drop in preference percentage (*p* < 0.0001) when compared to the control group. Similarly, when compared to zebrafish treated with Sco alone, these amnesic effects were dramatically reduced by exposure to sweroside, with *p* < 0.00001 for 2.79, 8.35, and 13.95 nM.

Our data are supported by the literature indicating that iridoids ameliorated cognitive impairment in AD models. Wang et al. [18] showed that cornuside, a secoiridoid glucoside, ameliorated memory impairments in Sco-induced AD mice, suggesting it to be a potential anti-AD candidate. Eskandarzadeh et al. [19] demonstrated that iridoid glycosides, including loganin, secologanin, and loganetin, improved memory impairment by the inhibition of glycogen synthase kinase-3β (GSK-3β).

### 2.3. Effects on AChE Activity

**Figure 4:**
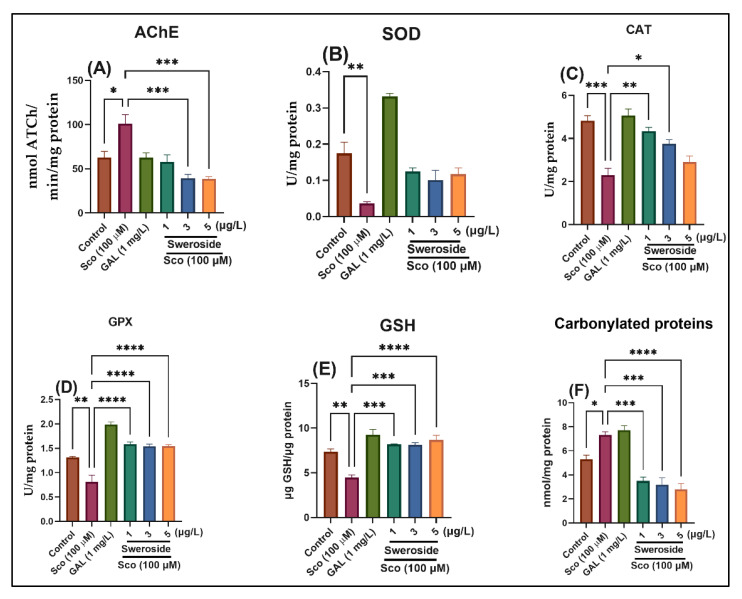
Effects of sweroside (2.79, 8.35, and 13.95 nM) on (**A**) AChE; (**B**) Superoxide dismutase (SOD); (**C**) Catalase (CAT); (**D**) glutathione peroxidase (GPX)-specific activities; (**E**) Reduced glutathione (GSH) and (**F**) carbonylated protein levels. Data are represented by means ± S.E.M. (*n* = 10 animals per group). * *p* < 0.01, ** *p* < 0.001, *** *p* < 0.0001, and **** *p* < 0.00001 (Tukey’s post hoc analyses). Galantamine (GAL, 2.71 μM) was used as a reference positive drug.

Because the Sco-induced zebrafish model was connected to the cholinergic injury, we investigated how the cholinergic system worked in the Sco zebrafish brain. In Figure 4A, the AChE activity is displayed. One-way ANOVA revealed the treatment’s overall significant impact on AChE activity [F (5, 54) = 11.60] (*p* < 0.0001). When compared to the control group, the Sco treatment significantly (*p* < 0.01) boosted the AChE activity in the zebrafish brain. Conversely, sweroside treatment decreased AChE activity with *p* < 0.0001 for 8.35 nM and 13.95 nM compared to zebrafish receiving Sco alone. Our findings showed that in the Sco zebrafish brain, sweroside had a larger effect on cholinergic neurotransmission.

### 2.4. Effects on SOD-, CAT-, and GPX-Specific Activities

Considering that oxidative stress significantly influences the cognitive status in AD, the effects of sweroside on the antioxidant enzyme activities were investigated as a consequence. The significant overall effects of the treatment on SOD activity [F (5, 54) = 5.87] (*p* < 0.01) (Figure 4B), CAT activity [F (5, 54) = 17.05] (*p* < 0.0001) (Figure 4C), and GPX activity [F (5, 54) = 21.27] (*p* < 0.0001) (Figure 4D) according to one-way ANOVA were evidenced.

When it comes to SOD-specific activity, Sco significantly decreased SOD activity in comparison to the control group (*p* < 0.0001). However, sweroside significantly increased SOD activity in comparison to animals treated with Sco alone (*p* < 0.00001) (Figure 4B). When compared to the control group, Sco substantially reduced CAT activity (*p* < 0.0001), but sweroside treatment significantly increased it (*p* < 0.0001) (Figure 4C). When compared to the control group, the GPX-specific activity was dramatically reduced after Sco treatment (*p* < 0.001) (Figure 4D). However, sweroside significantly increased it (*p* < 0.0001) (Figure 4D) when compared to zebrafish treated with Sco alone.

### 2.5. Effects on GSH and Carbonylated Protein Levels

As shown in Figure 4E, Sco-induced zebrafish had considerably lower levels of reduced GSH than the control group (*p* < 0.0001). Sweroside significantly raised the decreased GSH content in the zebrafish when compared to the zebrafish treated with Sco alone (*p* < 0.0001). When compared to the control group, the amount of carbonylated protein increased significantly after Sco treatment (*p* < 0.001) (Figure 4F), but it was significantly reduced by sweroside treatment (*p* < 0.0001) when compared to zebrafish treated with Sco alone.

Learning and memory skills are disrupted in both animals and humans when the central muscarinic acetylcholine receptor is blocked. Sco, an anticholinergic (muscarinic blocker), has been used as a powerful amnesic agent. When examined on the same clinical battery, Sco-induced cognitive deficits in young volunteers are identical to those seen in senile individuals or people with dementia. Loss of memory for recent (but not immediate) experiences was one such weakness in young individuals [20]. Numerous studies have shown that chronic Sco administration decreases antioxidant defenses by suppressing nuclear factor erythroid 2-related factor 2 (Nrf2) expression and is linked to decreased synaptic plasticity via the cAMP response element-binding protein (CREB)/BDNF pathway [21,22]. Furthermore, Sco-induced oxidative stress raises AChE activity and phosphorylates GSK, which eventually causes apoptosis through Bax/Bcl-2 mediators [23,24]. The synthesis and release of acetylcholine (ACh) were slowed in the brains of AD patients, while AChE was overactive and degraded more ACh, causing cholinergic system transmission to be hampered [25,26,27]. In the Sco-induced model, sweroside inhibited AChE activity, suggesting positive effects on cholinergic neurotransmission. Oxidative stress contributes to the evolution of AD by impairing brain tissue and causing degenerative changes [28]. Aged animal models showed considerable connections between the emergence of behavioral deficits or cognitive impairments in learning and memory retention, as well as temporal and spatial memory [29]. Carbonylated proteins are the products of protein oxidation [30], while SOD and CAT were considered to be two important antioxidant enzymes to drive off oxidative stress [31,32]. In the present study, the administration of sweroside reduced carbonylated protein levels by increasing the specific activities of SOD, CAT, and GPX in the zebrafish brain. Moreover, as an antioxidant agent, sweroside increased the total content of reduced GSH in the Sco-induced model, suggesting that sweroside could be considered a strong antioxidant agent.

## 3. Materials and Methods

### 3.1. Plant Material and Extraction Method

*Schenkia spicata* (Gentianaceae) aerial parts were collected From Bahariya Oasis, Giza, Egypt, in July 2020. The plant material was identified by Dr. Mohamed El-Gebali, former researcher of Botany at the National Research Centre (NRC) of Cairo, Egypt. A voucher specimen was kept at the Pharmacognosy Department, Faculty of Pharmacy, Ain Shams University, under accession number (PHG-P-SS-404).

Powdered plant material (2 kg) was macerated in aqueous methanol, 70% (3 × 3 L). The filtrates were collected, evaporated to dryness under vacuum, and subjected to freeze-drying to yield a yellow amorphous powder (70 g).

### 3.2. Isolation of Sweroside

The extract (30 g) was fractionated by column chromatography on Dia-ion HP-20 column (5 cm × 140 cm, 0.50 kg) using H_2_O and methanol (MeOH) mixtures in order of decreasing polarities (as an eluent to yield four main fractions (I–IV). Each fraction was examined by two-dimension paper chromatography using 6% acetic acid (HOAc) and butanol: acetic acid: water (BAW) as eluents.

Fractions II (17 g) was the major fraction applied to repeated Sephadex LH-20 (column using H_2_O/MeOH (1:1), followed by preparative paper chromatography (Whatman filter paper sheets 3MM) using 6% HOAc as an eluent to yield one major pure compound (5 g).

### 3.3. Structure Elucidation of Sweroside

Off-white powder of molecular ion [M-H]^−^ at *m*/*z* 357.

**Figure 5:**
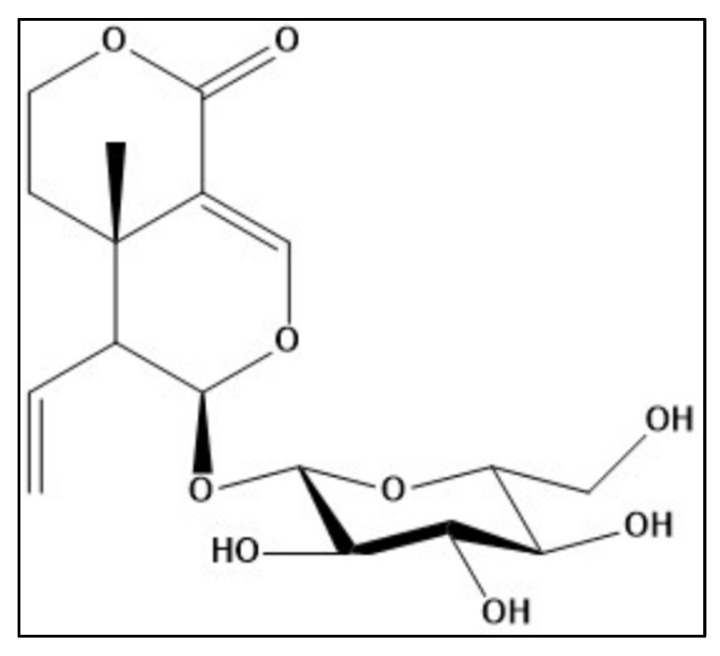
Chemical structure of sweroside.

^1^H-NMR (500 MHz, Acetone-*d*_6_): δ ppm: 5.46 (H-1, d, *J* = 1.62 Hz, 1H), 7.46 (H-3, *d*, *J* = 2.58 Hz, 1H), 3.10 (H-5, m, 1H), 1.74 (H-6a, m, 1H), 1.61 (H-6b, m, 1H), 4.34 (H-7a, m, 1H), 4.26 (H-7b, m, 1H), 5.53 (H-8, m, 1H), 2.65 (H-9, m, 1H), 5.29 (H-10a, *dd*, *J* = 17.22 &1.88 Hz, 1H), 5.23 (H-10b, *dd*, *J* = 10.12 &1.88 Hz), 4.68 (H-1’, d, *J* = 7.65 Hz, 1H), 3.10 to 3.3.4 (m, H-2’, H-3’ and H-4’), 3.65 (H-6’a), 3.84 (H-6’b) (Figure 5).

^13^C-NMR (125 MHz, Acetone-*d*_6_): δ ppm 98.50 (C-1), 151.53 (C-3), 105.32 (C-4), 28.84 (C-5), 24.52 (C-6), 67.92 (C-7), 132.63 (C-8), 42.45(C-9), 119.86 (C-10), 165.02 (C-11), 98.52 (C-1’), 73.61 (C-2’), 76.72(C-3’), 70.49 (C-4’), 77.11(C-5’), 61.90 (C-6’) [33].

### 3.4. Animals

In the animal facility of the Alexandru Ioan Cuza University of Iasi, Faculty of Biology, Romania, seventy adult zebrafish (*Danio rerio*) of the short-finned phenotype (50:50 sex ratio) were maintained in a recirculating system with dechlorinated and aerated water at a controlled temperature (26 °C ± 2) and photoperiod 14:10 h (light: dark cycle). The pH of the water was kept at 7.5, the dissolved oxygen level was 7.20 mg/L, the ammonium concentration was <0.004 ppm, and the conductivity was 500 μS. Fish were given Norwin Norvitall flake twice daily. Before the studies, the animals underwent one week of acclimation. The seven groups (*n* = 10 animals per group) employed were: control, Sco (100 µM), sweroside treatment (2.79, 8.35, and 13.95 nM), galantamine (GAL, 2.71 μM), and imipramine (IMP, 63.11 μM). Data on doses used were reported in full in a prior report [34]. Animals were randomly assigned to the experimental groups. Without attrition or exclusion, all examined animals were accounted for in the analyses. All trials were carried out according to plan, and all endpoints measured were considered in the analysis. Highly trained experimenters who were blind to the zebrafish treatment carried out behavioral evaluations. Statistical analyses of data were performed blind, and the findings were beyond the control of the experimenters. In addition, we confirm that *n* = 10 animals/group is appropriate using InVivoStat, and the R-based statistical package [35]. Based on a significance level of 0.05, the power to detect a 20% biologically relevant change is 98%. The zebrafish exposed to Sco received sweroside by immersion once daily for 8 days in a 500 mL glass for 1 h, while Sco was administered 30 min before the beginning of the behavioral assessment. The GAL and IMP were delivered into the Sco-treated fish 30 min before the behavioral testing. The data analysis and presentation, as well as the study’s experimental design, all followed the PREPARE and ARRIVE criteria [36] for planning and organizing animal testing and research, respectively. All experimental procedures were approved by the Ethics Committee on Animal Research of the Alexandru Ioan Cuza University of Iasi, Romania, Faculty of Biology (No. 15309/30.06.2019), and found that they complied fully with the Directive 2010/63/EU of the European Parliament and of the Council of 22 September 2010 on the protection of animals. Animal welfare was improved, and every effort was made to reduce the use of animals.

### 3.5. Novel Tank Diving Test (NTT)

Cachat et al. [37] described the NTT as a particular test for evaluating both locomotor activity and anxiety response in zebrafish. A 1.5 L trapezoidal tank (15.2 × 27.9 × 7.1 cm) was separated into top and bottom parts by a virtual horizontal line. The locomotion and exploratory behavior of individual zebrafish were tested for 6 min and analyzed using ANY-maze⁠^®^software (Stoelting Co., Wood Dale, IL, USA). The endpoints investigated were the total distance traveled (m) and average velocity (m/s) for assessing the locomotor activity and the number of entries to the top, time spent in the top (s), average entry duration (s), and freezing duration (s) for evaluating the anxiety-like behavior.

### 3.6. Y-maze Test

This test was used to assess zebrafish responses to novelty and locomotor activity [38]. Individual zebrafish were trained in a Y-shaped glass aquarium (3 L) with three arms (25 × 8 × 15 cm) designed as the “start” arm (always open), the “novel” arm (open during the test trial), and the permanently open arm. The fish were individually placed in the start arm, and the novel arm was closed for the initial training session (5 min). The second training session (5 min) started after 1 h, and the fish were placed in the start arm once more, with the novel arm opened. The time spent in the novel arm (% of total arm time) was used for assessing the response to novelty, whereas total distance traveled (m) and turn angle (°) were used for the locomotor activity characterization. Videos were analyzed by ANY-maze⁠^®^software (Stoelting Co., Wood Dale, IL, USA).

### 3.7. Novel Object Recognition Test (NOR)

To study the zebrafish memory ability, NOR was employed [39]. In the habituation phase, zebrafish were given 5 min of acclimatization to the novel tank (30 × 30 × 30 cm) in the absence of the objects for three days. Zebrafish were exposed to two similar objects for 10 min on the fourth day (training phase). One hour following the training phase, one of two similar objects (FO, familiar objects) was randomly changed with a novel object (NO), and the interaction was observed for 10 min (testing phase). The preference percentages were calculated as follows: [time of exploration of NO/time of exploration of FO + time of exploration of NO × 100]. The behavior was analyzed by ANY-maze⁠^®^software (Stoelting Co., Wood Dale, IL, USA).

### 3.8. Acetylcholinesterase (AChE), Antioxidant Enzyme, Glutathione (GSH), and Protein Carbonyl Measurment

Acetylcholinesterase (AchE)-, superoxide dismutase (SOD)-, catalase (CAT)-, and glutathione peroxidase (GPX)- specific activities as well as glutathione (GSH) and protein carbonyl levels were assessed using the procedures thoroughly outlined by Brinza et al. [40]. A bicinchoninic acid (BCA) protein assay kit (Sigma-Aldrich, Darmstadt, Germany) was used to assess the total protein concentration [41].

### 3.9. Statistical Analyses

The mean ± standard error of the mean (S.E.M) was used to express the results. GraphPad Prism 9.0 (GraphPad Software, Inc., San Diego, CA, USA) was used to conduct the statistical analyses, which included one-way analysis of variance (ANOVA) and the Tukey post hoc test for multiple comparisons. At *p* < 0.05, statistical significance was assumed to be present.

## 4. Conclusions

In conclusion, sweroside ameliorated Sco-induced anxiety and memory deficits in behavioral tests, including the NTT test, Y-maze, and NOR tests, which confirmed its therapeutic effect on the zebrafish model. The mechanism was to inhibit the AChE activity and brain oxidative stress, which finally contributed to cholinergic neurotransmission and cognitive improvement. In this study, sweroside was confirmed to be a potential antiamnesic candidate in zebrafish for the first time, providing more information for its further application in the treatment of dementia-related conditions.

## Data Availability

The data presented in this study are available on request from the corresponding author.

## Sample Availability

Not available.
